# Impact of the COVID-19 Pandemic on the Implementation and Adoption of a Virtual Fracture Clinic Pathway: A Closed Loop Audit

**DOI:** 10.7759/cureus.76317

**Published:** 2024-12-24

**Authors:** Daire-Sean Gibbons, Aaron A Glynn

**Affiliations:** 1 Department of Trauma and Orthopaedic Surgery, Our Lady of Lourdes Hospital Drogheda, Royal College of Surgeons in Ireland (RCSI) Hospital Group, Drogheda, IRL

**Keywords:** boast guidelines, covid-19 pandemic, early discharge, face-to-face fracture clinic, patient-initiated follow-up, virtual fracture clinic

## Abstract

Introduction: Trauma and orthopedics departments have traditionally used face-to-face (FTF) fracture clinics for non-operative fractures. Developed in 2011, the virtual fracture clinic (VFC) was fully implemented at an institution during the COVID-19 pandemic to reduce in-person interactions.

Aims: First, the study aims to measure the percentage of non-operative patients triaged through the VFC when this was optional and re-audit after implementing a COVID-19-related policy change mandating VFC triage. Second, the study aims to measure the number of FTF fracture clinic interactions and re-audit after implementing three policies: national COVID-19 lockdowns, mandated VFC triage, and early appropriate discharge.

Methods: Data from two periods were examined, pre-pandemic (2018-2020) and pandemic (2020-2022), at a university teaching hospital. We measured compliance with modified British Orthopaedic Association Standards for timely senior review, minimizing outpatient visits, and patient-initiated follow-up.

Results: The percentage of cases triaged to the VFC rose from 39% to 100%. FTF fracture clinic interactions dropped by 50.2% from 35,399 to 17,639. All three policy changes reduced FTF numbers: 3.7% due to national lockdowns, 14.7% due to VFC triage, and 35.5% due to early appropriate discharge.

Conclusions: The COVID-19 pandemic provided a window in which healthcare working partners were more receptive to change. Our institution successfully used this opportunity to implement policy changes that improved patient care and maximized resources.

## Introduction

Traditionally, trauma and orthopedics departments in the United Kingdom and Ireland have used a face-to-face (FTF) fracture clinic pathway. Patients with minor non-operative fractures are given a fracture clinic appointment by emergency department (ED) staff without an initial orthopedic review. Although this system appears to streamline treatment and follow-up, it has limitations, including a high number of attendance at FTF clinics and potential delays from initial management to appropriate follow-up [1].

The virtual fracture clinic (VFC) was a model for rapid orthopedic injury assessment developed at the Glasgow Royal Infirmary in 2011 [2]. In 2016, Tullamore's Department of Trauma and Orthopaedic Surgery in Ireland introduced a similar trauma assessment clinic (TAC) [3]. Both models emphasize the review of non-operative orthopedic injuries by a multidisciplinary team led by a senior orthopedic surgeon no more than 24 hours after initial treatment. After this VFC, patients are contacted by phone and informed of their care plan: immediate discharge to GP or physiotherapy, an FTF appointment at an appropriate interval, or immediate hospital admission for surgery if necessary.

Our Department of Trauma and Orthopaedics is based at a major teaching hospital in a developed European economy and is a referral center for four peripheral locations. The FTF fracture clinic represented the unit’s busiest outpatient service. After the successful implementation of the TAC in Tullamore, a similar model was trialed at our institution in 2017. However, the utilization of the VFC remained limited, and FTF clinic numbers stayed high.

Like many other trauma units, the COVID-19 pandemic prompted an immediate re-evaluation of the fracture care pathway at our institution to minimize unnecessary patient-staff interactions [4]. While major orthopedic injuries requiring operative intervention were still referred to the on-call service by telephone, all non-operative injuries were referred for review via a smartphone app (Siilo-Medical Messenger). After initial treatment advice, patients were allowed home from the ED and reviewed in the VFC within 24 hours [5]. The goal was to reduce in-person interactions by prioritizing virtual care and discharges [6].

This audit examined patient interactions in two two-year time periods: a pre-COVID-19 period and the COVID-19 pandemic period. We measured both the utilization of the VFC and the number of clinical interactions at the FTF fracture clinic. In the latter, we aimed to isolate the effect of three policies: national COVID-19 lockdowns, full implementation and mandating of the VFC pathway, and enhanced discharge from FTF clinics.

This work was previously presented at the Atlantic Orthopaedic Club Meeting on the 16th of November, 2024, and the Annual Scientific Meeting of the British Trauma Society on the 21st of November, 2024.

## Materials and methods

Study design and setting

This audit was conducted in a developed western European economy at a university teaching hospital, which also serves as a regional referral center for trauma and orthopedic services. The study analyzed patient data over two distinct two-year periods: the pre-pandemic period, spanning from March 2018 to February 2020, and the pandemic period, from March 2020 to February 2022. The analysis focused on understanding the impact of COVID-19-related policies on both the implementation of the VFC and FTF fracture clinic interactions. All data collection and analysis adhered to ethical guidelines and institutional policies for clinical audits.

Inclusion and exclusion criteria

The study included all patients referred to the trauma and orthopedic department for non-operative management during the designated timeframes. Referrals managed by locum consultants or cases requiring operative intervention at the initial referral stage were excluded from the analysis. This approach ensured that the study focused specifically on non-operative pathways relevant to the VFC model.

Data sources

Data were sourced from two primary systems routinely used for clinical operations. The Integrated Patient Management System (IPMS, Dedalus Group, Florence, Italy) provided detailed records of patient referrals, clinic appointments, and other interactions within the department. Additionally, data from the Department of Physiotherapy’s electronic records, maintained in Microsoft Excel (Microsoft Corporation, Redmond, WA, USA), offered information on therapy referrals and follow-up appointments. These comprehensive data sources enabled a thorough evaluation of patient pathways and adherence to the VFC model.

Audit standards and measurements

Three modified British Orthopaedic Association Standards (BOASTs) were used in this study. The implementation of the VFC pathway was measured against the BOAST standard for early senior review [5]. Compliance with these standards was measured through the percentage of patients reviewed virtually versus in person. The reduction in FTF interactions was measured against two BOAST standards for minimizing outpatient visits and patient-initiated follow-up [6].

VFC pathway

During the pandemic, all non-operative injuries were referred to the VFC via the Siilo-Medical Messenger smartphone application (Siilo Holdings B.V., Amsterdam, Netherlands). This digital platform streamlined communication between the orthopedic department and EDs at both the referral center and the four other peripheral sites. This enabled case discussions among clinicians and facilitated prompt triage to operative and non-operative care. The VFC model ensured that a multidisciplinary team led by a senior orthopedic consultant reviewed non-operative patients every morning from the previous 24-hour trauma cycle. Depending on the assessment, patients were either discharged, referred to physiotherapy, referred to an FTF clinic, or, if necessary, recalled for immediate operative care.

Data analysis

The analysis focused on several key parameters, including the distribution of clinic appointments between FTF and virtual formats, trends in orthopedic referrals, and outcomes related to compliance with the BOASTs. Reductions in FTF appointments were attributed to three primary factors: national COVID-19 lockdown policies, changes in triage protocols/VFC, and enhanced discharge policies. Data analysis was performed using Microsoft Excel, with percentages and absolute changes calculated to evaluate the effectiveness of the VFC pathway.

Quality control

Several quality control measures were implemented to maintain the accuracy and integrity of the data. Data were extracted directly from the IPMS and the Department of Physiotherapy’s electronic records, both of which are routinely maintained and updated for clinical use. The inclusion and exclusion criteria were clearly defined and consistently applied to ensure that the study remained focused on non-operative trauma referrals. Key metrics, such as the number of virtual and FTF appointments, were collectively reviewed by the audit team to verify that findings aligned with expectations based on departmental records and operational changes. Additionally, trends in referral and appointment data across the study periods were compared against institutional reports to validate consistency and identify any significant discrepancies. These measures ensured that the findings accurately represented clinical practices and outcomes during the study periods.

## Results

**Figure 1:**
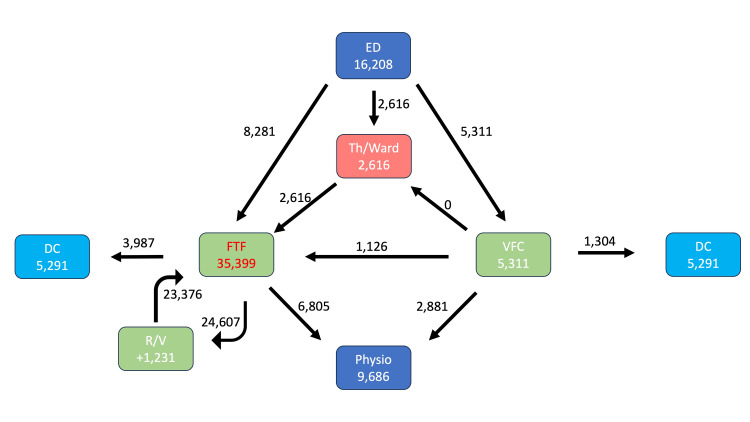
Pre-COVID-19 pathway The patient pathway for trauma orthopedics is shown for the pre-COVID-19 period. Numbers represent the total number of patients through each part of the pathway for the total period of 23 months. ED: emergency department, Th: theatre, DC: discharge, FTF: face-to-face, VFC: virtual fracture clinic, R/V: review, COVID-19: coronavirus disease 2019

**Figure 2:**
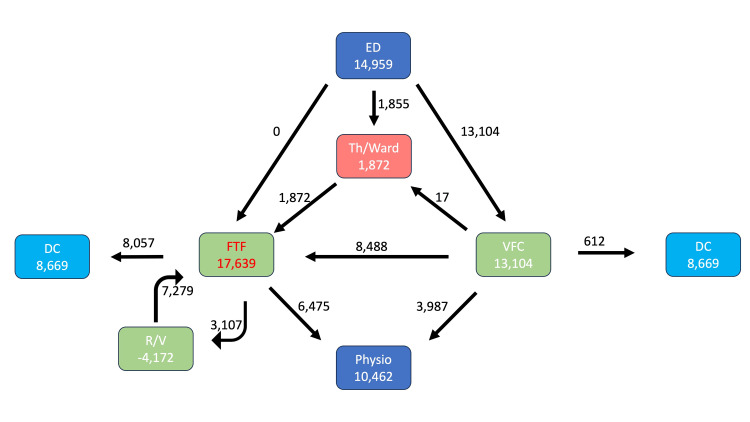
COVID-19 pathway The patient pathway for trauma orthopedics is shown for the COVID-19 period. Numbers represent the total number of patients through each part of the pathway for the total period of 23 months. ED: emergency department, Th: theatre, DC: discharge, FTF: face-to-face, VFC: virtual fracture clinic, R/V: review

**Figure 3:**
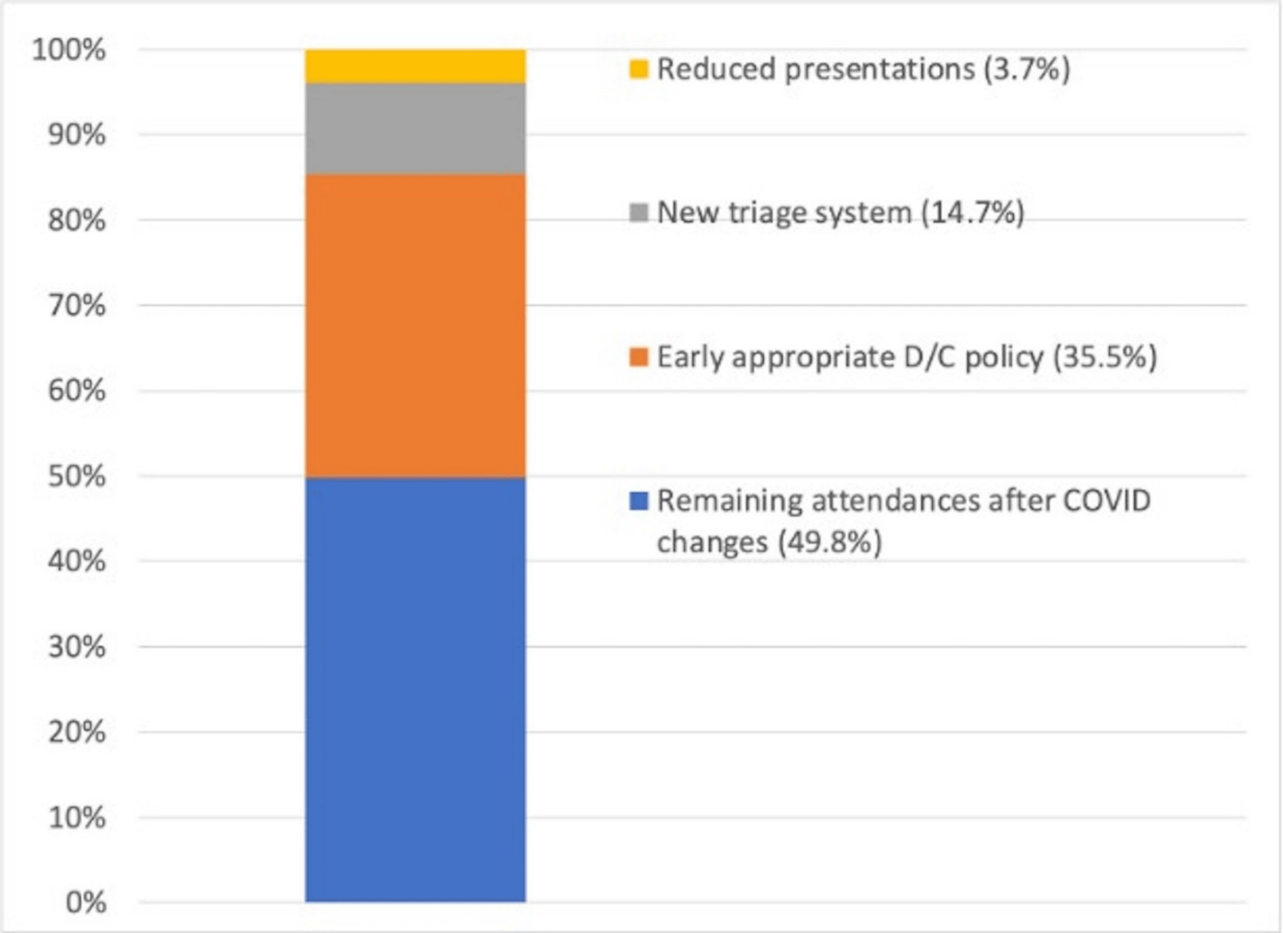
Sources of reductions in FTF clinic attendance During the pre-COVID-19 period, FTF clinic attendance numbered 35,339 (100%). During the COVID-19 period, this was only 17,639 (49.8%) due to three factors: reduced orthopedic referrals (3.7%), changes in orthopedic triage/use of VFC (14.7%), and early appropriate DC from FTF clinics (35.5%). DC: discharge, FTF: face-to-face, VFC: virtual fracture clinic, COVID-19: coronavirus disease 2019

As shown in Figure 1 and Figure 2, pre-pandemic orthopedic referrals totaled 16,208, which dropped to 14,959 during the COVID-19 pandemic. The percentage of non-operative cases triaged to the VFC increased from 39% to 100%. FTF clinic appointments dropped from 35,339 to 17,639 due to 3 factors: reduced orthopedic referrals/national COVID-19 lockdowns, implementation of the VFC, and early appropriate discharge from FTF clinics, as shown in Figure 3.

Standard 1

Following acute traumatic orthopedic injury, the BOAST guideline recommends that patients be seen in a new fracture clinic within 72 hours of presenting the injury [5]. Our measure compared the percentage of patients initially reviewed in a VFC within 24 hours versus an FTF clinic. During the pre-pandemic period, 39% of all orthopedic non-operative patients were triaged to the VFC for assessment within 24 hours, while 61% were triaged directly to the FTF clinic, potentially causing delays. In contrast, during the re-audit period, 100% of non-operative patients were assessed in the VFC within 24 hours after initial treatment in the emergency department.

Standard 2

During the coronavirus pandemic, there was an increased emphasis on managing patients with non-operative strategies and minimizing outpatient visits [6]. As part of this effort, changes in triage protocols and the introduction of VFC led to a significant reduction in FTF attendance. Prior to the pandemic, there were 35,399 FTF fracture clinic appointments, which decreased to 17,639 during the COVID-19 pandemic, a reduction of 50.2%. Of this, national COVID-19 lockdown policies and associated reduced activity accounted for only 3.7%, while the change in triage protocols and VFC implementation contributed to a 14.7% reduction.

Standard 3

BOAST emphasizes that "patient-initiated follow-up should be the default" [6]. In alignment with this principle, our measure focused on reducing FTF attendance by implementing an early and appropriate discharge policy. The results demonstrated a significant impact, with a 35.5% reduction in FTF attendance compared to pre-pandemic levels.

## Discussion

This audit revealed significant changes in patient pathways between the pre-pandemic and pandemic periods. Despite Ireland's stringent lockdown policies, only a small portion (3.7%) of the reduction in FTF clinic attendance was attributable to reduced societal activity during lockdowns [7]. The effect of lockdowns on trauma presentations is complex. Early lockdowns were associated with increased physical activity levels [8]. At the same time, later periods saw a "rebound effect," where individuals, having become sedentary during the lockdown, resumed vigorous physical activity, leading to increased trauma presentations [9,10]. However, most of the changes were due to institutional policy shifts, particularly the full implementation of the VFC and enhanced discharge policies. These improvements align with similar findings in the literature, as outlined below.

Timeliness of care and triage efficiency (BOAST standard 1)

Our audit demonstrated a significant improvement in the timeliness of care, with 100% of non-operative cases reviewed within 24 hours during the pandemic. This finding mirrors Murphy et al.’s review, which emphasized the efficiency gains from VFCs [2]. However, few studies specifically examined the time required for senior assessment. McKirdy and Imbuldeniya reported a marked increase in compliance with BOAST's 72-hour senior review standard, rising from 5.1% to 46.4% [11]. In comparison, Holgate et al. reported compliance jumping from 6% to 100%, with an average assessment time of 1.3 days [12].

Reduction in FTF clinic attendance (BOAST standard 2)

VFC triage alone contributed to our audit's 14.7% reduction in FTF appointments. This is similar to the 31.5% decrease in new FTF appointments reported by O’Reilly et al. [3]. More dramatic reductions have been observed in other studies. McKirdy and Imbuldeniya documented a 72.2% reduction in new FTF clinics [11], while Brogan et al. reported an 87% reduction in FTF clinics for fifth metatarsal fractures [13]. Similarly, Ferguson et al. showed a decrease in FTF visits for new patients from 96.8% to 19% [14]. Despite variations, these studies consistently demonstrate that VFC models significantly reduce unnecessary clinic visits and optimize resource use.

Patient-initiated follow-up and appropriate discharge (BOAST standard 3)

Early discharge policies and patient-initiated follow-up led to a 35.5% reduction in FTF attendance in our audit. While Murphy et al. [2] noted reductions in FTF clinic visits with VFCs, most studies focus on new patients and do not isolate the effects of discharge and follow-up policies. Early discharge and novel follow-up strategies have shown success in other healthcare settings. For example, Harrison et al. demonstrated that post-discharge telephone follow-up reduced hospital readmissions [15], while Abed et al. found that early discharge following hand surgery significantly reduced routine follow-ups [16]. These findings reinforce the effectiveness of early discharge and patient-initiated follow-up in reducing unnecessary follow-up visits and enhancing clinic efficiency.

Limitations

A key limitation of our study is that it did not assess patient safety or satisfaction, both of which are critical for evaluating the overall success of VFCs. However, other studies have addressed these factors. For example, Murphy et al. reported high patient satisfaction and no adverse outcomes with VFCs [2], while O'Reilly et al. found that VFCs improved satisfaction by reducing wait times and increasing convenience [3]. Future research at our institution could explore these aspects. Additionally, while VFCs have been highly effective during the pandemic, their success in non-pandemic times may be limited as demand for in-person care increases.

## Conclusions

Busy hospital services such as trauma and orthopedics require efficiency to provide good patient care. The first round of our audit revealed both limited uptake of the VFC pathway and high FTF clinic numbers. During the COVID-19 pandemic, national and local policy changes were introduced, and our service was re-audited. Mandated triage to VFC was successfully implemented with 100% compliance. FTF clinic numbers halved. Only a small reduction was due to national COVID-19 lockdown policies and reduced activity. Most of the reduction was due to the VFC triage policy and the early appropriate discharge policy. The results of this study will be of interest to other clinicians and administrators as they weigh the potential costs and benefits of similar policy changes at their own institutions.
